# Parliament and Publications

**Published:** 1911-04-01

**Authors:** 


### Parliament and Publications.

### SICKNESS AMONG PAUPERS.

The immense mass of statistics, compiled at the instance of the Poor Law Commission and contained in a bulky Blue-Book which has just been issued, is so well arranged that there is little difficulty in sifting out the figures dealing directly with sickness among paupers. The regular statistics of pauperism give very little information as to sickness among the persons relieved. Accordingly a return was obtained in April 1907 from 128 unions in England and Wales showing the number of paupers (including casuals) under treatment by the medical officers at noon on Saturday, April 13, 1907. The inquiry extended to an area comprising a population of 10,071,347, or 31.0 per cent. of the population of England and Wales. The returns received related to 249,901 paupers, or 30.0 per cent. of the number chargeable on January 1, 1907 (excluding lunatics in asylums, registered hospitals, or licensed houses). The number of persons receiving medical treatment at the cost of the rates was found to be 78,511, or 31.4 per cent. of the total number returned. The total receiving treatment comprised 30,497 men, 37,244 women, and 10,770 children under sixteen years of age. In every group the proportions under medical treatment were higher among indoor paupers than among outdoor paupers. The highest proportion occurred among the men under fifty years of age, of whom nearly two out of every three were suffering from sickness of some form. Analysing the cases according to their character it is found that upwards of one half are medical cases of an acute or chronic nature, and that nearly one-half of these cases are relieved in their own homes. Of the 51,033 patients in Poor Law institutions, 23,507 were under treatment in workhouses, 17,814 in separate infirmaries, and 9,712 in other Poor Law institutions. The following points appear to be established: (1) Nearly one-third of the persons in receipt of relief are under medical treatment. (2) This proportion rises to nearly one-half in the case of indoor poor, and falls to one-fifth in the case of outdoor poor. (3) Over three-fourths of the paupers under medical treatment in London are relieved in Poor Law institutions, while less than one-half are so relieved outside London. (4) The London Poor Law infirmaries attract considerable numbers of the young and those of middle age. (5) The five most important groups of the specified diseases among the persons relieved and the proportions under medical treatment for each of them are: (*a*) Bronchitis and pneumonia, 44.0 per 1,000; (*b*) rheumatism and gout, 19.9 per 1,000; (*c*) pulmonary tuberculosis, 15.8 per 1,000; (*d*) heart disease, 13.9 per 1,000; (*e*) ulcerated legs, 12.1 per 1,000. (6) Calculated upon the proportions shown by the Return it would appear that the number of pauper epileptics in the whole of England and Wales not certified as insane is probably about 5,500. The Blue-Book also contains reports upon insurance against invalidity, sickness, etc., by Mr. T. G. Ackland, Mr. G. King, and Mr. F. G. P. Neilson. "Royal Commission on the Poor Laws," Appendix volume xxv., Cd. 5077, price 11s. 1d.

